# Chemical Pesticides and Human Health: The Urgent Need for a New Concept in Agriculture

**DOI:** 10.3389/fpubh.2016.00148

**Published:** 2016-07-18

**Authors:** Polyxeni Nicolopoulou-Stamati, Sotirios Maipas, Chrysanthi Kotampasi, Panagiotis Stamatis, Luc Hens

**Affiliations:** ^1^Department of Pathology, School of Medicine, National and Kapodistrian University of Athens, Athens, Greece; ^2^Vlaamse Instelling voor Technologisch Onderzoek (VITO), Mol, Belgium

**Keywords:** pesticides, agrochemicals, environmental health, endocrine disruptors, food sovereignty

## Abstract

The industrialization of the agricultural sector has increased the chemical burden on natural ecosystems. Pesticides are agrochemicals used in agricultural lands, public health programs, and urban green areas in order to protect plants and humans from various diseases. However, due to their known ability to cause a large number of negative health and environmental effects, their side effects can be an important environmental health risk factor. The urgent need for a more sustainable and ecological approach has produced many innovative ideas, among them agriculture reforms and food production implementing sustainable practice evolving to food sovereignty. It is more obvious than ever that the society needs the implementation of a new agricultural concept regarding food production, which is safer for man and the environment, and to this end, steps such as the declaration of Nyéléni have been taken.

## Introduction

Pesticides are substances or mixtures of substances that are mainly used in agriculture or in public health protection programs in order to protect plants from pests, weeds or diseases, and humans from vector-borne diseases, such as malaria, dengue fever, and schistosomiasis. Insecticides, fungicides, herbicides, rodenticides, and plant growth regulators are typical examples (1–3). These products are also used for other purposes, such as the improvement and maintenance of non-agricultural areas like public urban green areas and sport fields (4, 5). Furthermore, there are other less known applications of these chemical substances, such as in pet shampoos (4), building materials, and boat bottoms in order to eliminate or prevent the presence of unwanted species (6).

Many of the pesticides have been associated with health and environmental issues (1, 2, 7–12), and the agricultural use of certain pesticides has been abandoned (2). Exposure to pesticides can be through contact with the skin, ingestion, or inhalation. The type of pesticide, the duration and route of exposure, and the individual health status (e.g., nutritional deficiencies and healthy/damaged skin) are determining factors in the possible health outcome. Within a human or animal body, pesticides may be metabolized, excreted, stored, or bioaccumulated in body fat (1, 2, 13). The numerous negative health effects that have been associated with chemical pesticides include, among other effects, dermatological, gastrointestinal, neurological, carcinogenic, respiratory, reproductive, and endocrine effects (1, 2, 8, 10, 14–30). Furthermore, high occupational, accidental, or intentional exposure to pesticides can result in hospitalization and death (1, 31).

Residues of pesticides can be found in a great variety of everyday foods and beverages, including for instance cooked meals, water, wine, fruit juices, refreshments, and animal feeds (32–39). Furthermore, it should be noted that washing and peeling cannot completely remove the residues (40). In the majority of cases, the concentrations do not exceed the legislatively determined safe levels (36, 39, 41, 42). However, these “safe limits” may underestimate the real health risk as in the case of simultaneous exposure to two or more chemical substances, which occurs in real-life conditions and may have synergistic effects (1, 43). Pesticides residues have also been detected in human breast milk samples, and there are concerns about prenatal exposure and health effects in children (13, 44–46).

This current review aims at highlighting the urgent need for a new concept in agriculture involving a drastic reduction in the use of chemical pesticides. Given the fact that the health effects have been extensively discussed in the current literature, this paper focuses on the major chronic health effects and recent findings regarding health effects that have been associated with exposure to common classes of chemical pesticides, i.e., organochlorines, organophosphates, carbamates, pyrethroids, triazines, and neonicotinoids. More emphasis is given to the widely used herbicide “glyphosate,” which is an organophosphate pesticide very closely related to current agriculture (47). The important health effects, as discussed below, reveal the urgent need for implementing alternative solutions.

## Organochlorine Pesticides

The most widely known organochlorine pesticide is dichlorodiphenyltrichloroethane, i.e., the insecticide DDT, the uncontrolled use of which raised many environmental and human health issues (2, 48, 49). Dieldrin, endosulfan, heptachlor, dicofol, and methoxychlor are some other organochlorines used as pesticides.

There are a few countries that still use DDT or plan to reintroduce it for public health purposes (13, 48, 49). Furthermore, DDT is also used as a solution in certain solvents (2). It is a ubiquitous chemical substance, and it is believed that every living organism on Earth has a DDT body burden, mainly stored in the fat (48, 50). There is also evidence that DDT and its metabolite p,p-dichlorodiphenyldichloroethylene (DDE) may have endocrine-disrupting potential and carcinogenic action (48). *In utero* exposure to both DDT and DDE has been associated with neurodevelopmental effects in children (51). Moreover, a recent study related DDE to hepatic lipid dysfunction in rats (50).

The general class of organochlorine pesticides has been associated with health effects, such as endocrine disorders (10, 52), effects on embryonic development (53), lipid metabolism (54), and hematological and hepatic alterations (55). Their carcinogenic potential is questioned, but concerns about possible carcinogenic action should not be underestimated (38, 39, 56, 57).

## Organophosphorus Pesticides

Organophosphates, which were promoted as a more ecological alternative to organochlorines (58), include a great variety of pesticides, the most common of which is glyphosate. This class also includes other known pesticides, such as malathion, parathion, and dimethoate; some are known for their endocrine-disrupting potential (10, 59, 60). This class of pesticides has been associated with effects on the function of cholinesterase enzymes (58), decrease in insulin secretion, disruption of normal cellular metabolism of proteins, carbohydrates and fats (54), and also with genotoxic effects (61) and effects on mitochondrial function, causing cellular oxidative stress and problems to the nervous and endocrine systems (54).

Population-based studies have revealed possible relations between the exposure to organophosphorus pesticides and serious health effects including cardiovascular diseases (62), negative effects on the male reproductive system (63) and on the nervous system (58, 64–66), dementia (67), and also a possible increased risk for non-Hodgkin’s lymphoma (68). Furthermore, prenatal exposure to organophosphates has been correlated with decreased gestational duration (69) and neurological problems occurring in children (70).

Regarding glyphosate, the safety of which is the subject of an ongoing scientific controversy (60, 71–76), it is the most widely used herbicide in current agriculture (47, 75), especially since the introduction of glyphosate-tolerant genetically modified crops, such as certain types of soybean and maize (60, 77–80). Its extensive use in genetically modified soybean cultivation has raised concerns about possible synergistic estrogenic effects due to the simultaneous exposure to glyphosate and to the phytoestrogen “genistein,” which is a common isoflavone present in soybeans and soybean products (80, 81).

Glyphosate can display endocrine-disrupting activity (80, 82), affect human erythrocytes *in vitro* (83), and promote carcinogenicity in mouse skin (84). Furthermore, it is considered to cause extreme disruption in shikimate pathway, which is a pathway found in plants and bacteria as well as in human gut bacteria. This disruption may affect the supply of human organism with essential amino acids (85). Commercial glyphosate formulations are considered to be more toxic than the active substance alone (80, 83, 86, 87). Glyphosate-based herbicides, such as the well-known “Roundup,” can cause DNA damages and act as endocrine disruptors in human cell lines (60) and in rat testicular cells (88), cause damages to cultured human cutaneous cells (89), and promote cell death in the testicular cells of experimental animals (88, 90). There is evidence also for their possible ability to affect cytoskeleton and intracellular transport (91).

A recent study examined the possible relation between glyphosate, genetically modified crops, and health deterioration in the USA. Correlation analyses raised concerns about possible connections between glyphosate use and various health effects and diseases, such as hypertension, diabetes, strokes, autism, kidney failure, Parkinson’s and Alzheimer’s diseases, and cancer (82). Furthermore, there are concerns about the possible ability of glyphosate to cause gluten intolerance, a health problem associated with deficiencies in essential trace metals, reproductive issues, and increased risk to develop non-Hodgkin’s lymphoma (92).

## Carbamate Pesticides

Carbamate pesticides, such as aldicarb, carbofuran, and ziram, are another class of chemical pesticides that have been associated with endocrine-disrupting activity (10, 93), possible reproductive disorders (63, 93), and effects on cellular metabolic mechanisms and mitochondrial function (54). Moreover, *in vitro* studies have revealed the ability of carbamate pesticides to cause cytotoxic and genotoxic effects in hamster ovarian cells (94) and to induce apoptosis and necrosis in human immune cells (95), natural killer cells (96, 97), and also apoptosis in T lymphocytes (98).

Furthermore, it has been confirmed that carbaryl, which belongs to the category of carbamate pesticides, can act as a ligand for the hepatic aryl hydrocarbon receptor, a transcription factor involved in the mechanism of dioxin toxicity (99). There is also evidence for the ability of carbamate pesticides to cause neurobehavioral effects (65, 100), increased risk for dementia (67), and non-Hodgkin’s lymphoma (101).

## Other Classes of Chemical Pesticides

Triazines, such as atrazine, simazine, and ametryn, are another class of chemical pesticides that have been related to endocrine-disrupting effects and reproductive toxicity (10, 102, 103). Moreover, it was found that there is a possible statistical relationship between triazine herbicides and breast cancer incidence (104). Atrazine is the most known of the triazines, and it is a very widely used herbicide that has been associated with oxidative stress (103), cytotoxicity (105, 106), and dopaminergic effects (107, 108). Furthermore, the exposure of experimental animals to atrazine has been associated with reproductive toxicity (109) and delays in sexual maturation (110).

Synthetic pyrethroids, such as fenvalerate, permethrin, and sumithrin, are considered to be among the safer insecticides currently available for agricultural and public health purposes (111, 112). However, there is evidence for their ability to display endocrine-disrupting activity (10, 113–115), and to affect reproductive parameters in experimental animals including reproductive behavior (114, 116). Furthermore, a recent study related more than one pyrethroid metabolite to DNA damages in human sperm, raising concerns about possible negative effects on human reproductive health (117). It should also be mentioned that there are also concerns about their possible ability to display developmental neurotoxicity (25, 118, 119).

Neonicotinoid pesticides, such as imidacloprid, thiacloprid, and guadipyr, are relatively new and also the most extensively used insecticides (120) that were promoted for their low risk for non-target organisms (121). However, there is plenty of evidence to the contrary (115, 122–125); their effect on bees is a common example (124, 125). There is also evidence for possible effects on the endocrine and reproductive systems of animals (115, 126, 127). Moreover, a recent study demonstrated that neonicotinoids are able to increase the expression of the enzyme aromatase, which is engaged in breast cancer and also plays an important role during developmental periods (128).

## Urgent Need toward Cleaner and Safer Agricultural Practices

Current agricultural practices include the wide production and extensive use of chemicals known for their ability to cause negative health effects in humans and wildlife and to degrade the natural environment. Therefore, an urgent strategic approach is needed for a reduction in the use of agrochemicals and for the implementation of sustainable practices. Furthermore, current agriculture has to implement environmentally friendlier practices that pose fewer public health risks. Reforming agricultural practices aligned to fulfill these criteria is a step toward the sustainability of the agricultural sector in contrast to precision agriculture (129–134).

However, the reduction in the use of agrochemicals by applying them only when and where they are necessary, the spatiotemporal variability of all the soil and crop factors of a given field must be taken into consideration. This variability includes yield, field, soil, and crop variability but also factors, such as wind damage or flooding. Technological systems, such as geographical information systems, global positioning systems, and various sensors, can be useful (130–132, 135). These technological systems are developed by precision agriculture which of course we do not endorse, but we consider that selected technological tools can be used to decrease risks for environmental pollution and water pollution and to enhance economic benefits stemming from the reduction in the use of chemical products (130, 132).

It should be clear that the reform into an aggregate of machine-centered procedures and losing a human-centered character are not the desired. In contrast, the reduction in the use of pesticides assisted by innovative technological methods we strongly believe that may reduce the use of chemical substances or maybe it can lead to a total abandonment in many cases, such as in the case of urban green areas. The decision of the Italian village of Mals near the Austrian and Swiss borders to ban the use of pesticides and produce pesticide-free foods can be considered as a pioneer example across Europe. In 2014, more than 70% of the inhabitants of Mals who participated in a referendum voted against the use of pesticides (136). This historical decision apart that is consistent with the food sovereignty concept, which is discussed in the following section, also declares the need for disseminating information for raising awareness of the public in order to develop informed consents.

An innovative idea developed by the international movement “Via Campesina,” was the democratic concept of food sovereignty that has accompanied the progress toward sustainability for more than 20 years. It acquired a strong basis in 2007 in the African village Nyéléni in Mali, where representatives from more than eighty countries adopted the “Declaration of Nyéléni.” According to its principles, all the people of the world have the right to choose their own national and local policies to eliminate poverty, malnutrition, and hunger, to protect their traditions and also the natural environment (137–141).

The industrialization of agriculture has brought a series of problems including economic, social, and environmental impacts that local populations cannot manage. Furthermore, the overproduction of food, export-oriented monocultures, the demand for cheap labor, and the other characteristics of industrialization have clearly failed to solve the problems of hunger and malnutrition. On the contrary, inequitable food distribution, overexploitation of land and water sources, the overuse of agrochemicals, and the degradation of the natural environment are some of the results of the dominant agricultural model (138, 142–144). Food sovereignty promotes social, economic, and environmental sustainability, for instance, through the protection of the indigenous population and the production of food for distribution in local markets, and there is an ongoing effort for its recognition as a basic human right (138–140, 142, 145).

The dominant agricultural model has increased the chemical burden on natural environment (140, 142). Moreover, international agrochemical companies absorb traditional agricultural companies, leading to an industrialized agriculture model and leaving the local farmers and small producers to face the consequences (138, 143). In many cases, these people are obliged to adopt environmentally unfriendly techniques to increase their production in order to survive in the market, causing more environmental degradation (138). However, due to the fact that food sovereignty does not necessarily mean pesticide-free, organic food production, and because it does not determine pesticide use levels, for this reason, international eco-friendly standards should be implemented. People must be free to decide the method of production of their own food, and an important component of this decision concerns agrochemical products. The decision of the people of Mals to reject pesticides can be considered a step in this direction.

## Discussion

The need for protection against pests is a given and has its roots in antiquity, when both organic and chemical substances were applied as pesticides (146). Since then, numerous chemical pesticides have been produced, and now multinational agrochemical companies, which mostly control global food production, apply new chemical substances with pesticide properties and implement biotechnological advances, thus diverging from traditional agricultural methods. Furthermore, current agricultural practices are based on the wide use of chemical pesticides that have been associated with negative impacts on human health, wildlife, and natural environment (9, 11, 120, 147, 148).

Current agriculture has to deal with important factors, such as population growth, food security, health risks from chemical pesticides, pesticide resistance, degradation of the natural environment, and climate change (149–155). In recent years, some new concepts regarding agriculture and food production have appeared. A concept as such is climate-smart agriculture that seeks solutions in the new context of climate change (152, 153). Another major ongoing controversy exists between the advocates and the opponents of genetically engineered pesticide-resistant plants, regarding not only their safety (29, 156, 157) but also their impact on pesticide use (158–160).

Furthermore, the real-life chronic exposure to mixture of pesticides with possible additive or synergistic effects requires an in depth research. The underlying scientific uncertainty, the exposure of vulnerable groups and the fact that there are numerous possible mixtures reveal the real complex character of the problem (161–163). The combination of substances with probably carcinogenic or endocrine-disrupting effects may produce unknown adverse health effects. Therefore, the determination of “safe” levels of exposure to single pesticides may underestimate the real health effects, ignoring also the chronic exposure to multiple chemical substances.

Taking into consideration the health and environmental effects of chemical pesticides, it is clear that the need for a new concept in agriculture is urgent. This new concept must be based on a drastic reduction in the application of chemical pesticides, and can result in health, environmental, and economic benefits (164) as it is also envisaged in European Common Agricultural Policy (CAP) (165).

We believe in developing pesticide-free zones by implementing a total ban at local level and in urban green spaces is easily achievable. Furthermore, alternative procedures to the current model of food production should be implemented in new agricultural policies targeting sustainable development and protection of the consumers’ health. Despite the difficulties of establishing an innovative concept, the transition to a new cleaner and safer agricultural model is necessary.

## Author Contributions

Professor PN-S and Professor LH are the principal authors. Mr. SM contributed with proof reading, literature review, and editing. Mrs. CK and Mr. PS contributed with literature review and editing.

## Conflict of Interest Statement

The authors declare that the research was conducted in the absence of any commercial or financial relationships that could be construed as a potential conflict of interest.

## References

[1] World Health Organization. Public Health Impact of Pesticides Used in Agriculture. England: World Health Organization (1990).

[2] Alewu B Nosiri C Pesticides and human health. In: Stoytcheva M, editor. Pesticides in the Modern World – Effects of Pesticides Exposure. InTech (2011). p. 231–50. Available from: http://www.intechopen.com/books/pesticides-in-the-modern-world-effects-of-pesticides-exposure/pesticide-and-human-health

[3] NSW EPA. What Are Pesticides and How Do They Work? (2013). Available from: http://www.epa.nsw.gov.au/pesticides/pestwhatrhow.htm

[4] Hoffman RS Capel PD Larson SJ Comparison of pesticides in eight U.S. urban streams. Environ Toxicol Chem (2000) 19:2249–58. 10.1002/etc.5620190915

[5] Canadian Cancer Society. Cosmetic Pesticides. Information Brief. (2013). Available from: https://www.cancer.ca/~/media/cancer.ca/AB/get%20involved/take%20action/CosmeticPesticides-InformationBrief-AB.pdf

[6] Johnston JJ Introduction to Pesticides and Wildlife. USDA National Wildlife Research Center – Staff Publications Paper 589 (2001). Available from: http://digitalcommons.unl.edu/icwdm_usdanwrc/589

[7] Hayes TB Case P Chui S Chung D Haeffele C Haston K Pesticide mixtures, endocrine disruption, and amphibian declines: are we underestimating the impact? Environ Health Perspect (2006) 114:40–50. 10.1289/ehp.8051 16818245 PMC1874187

[8] Sanborn M Kerr KJ Sanin LH Cole DC Bassil KL Vakil C. Non-cancer health effects of pesticides. Systematic review and implications for family doctors. Can Fam Physician (2007) 53:1712–20. 17934035 PMC2231436

[9] Pimentel D Burgess M Environmental and economic costs of the application of pesticides primarily in the United States. In: Pimentel D Peshin R, editors. Integrated Pest Management. New York, Heidelberg, Dordrecht, London: Springer Science + Business Media Dordrecht (2014). p. 47–71.

[10] Mnif W Hassine AIH Bouaziz A Bartegi A Thomas O Roig B. Effect of endocrine disruptor pesticides: a review. Int J Environ Res Public Health (2011) 8:2265–2203. 10.3390/ijerph8062265 21776230 PMC3138025

[11] Goulson D Ecology: pesticides linked to bird declines. Nature (2014) 511:295–6. 10.1038/nature13642 25030159

[12] Zheng S Chen B Qiu X Chen M Ma Z Yu X Distribution and risk assessment of 82 pesticides in Jiulong River and estuary. Chemosphere (2016) 144:1177–92. 10.1016/j.chemosphere.2015.09.050 26461443

[13] Pirsaheb M Limoee M Namdari F Khamutian R. Organochlorine pesticides residue in breast milk: a systematic review. Med J Islam Repub Iran (2015) 29:228. 26478886 PMC4606957

[14] Semchuk KM Love EJ Lee RG Parkinson’s disease and exposure to agricultural work and pesticide chemicals. Neurology (1992) 42:1328–35. 10.1212/WNL.42.7.1328 1620342

[15] Wesseling C McConnell R Partanen T Hogstedt C. Agricultural pesticide use in developing countries: health effects and research needs. Int J Health Serv (1997) 27:273–308. 10.2190/E259-N3AH-TA1Y-H591 9142603

[16] Eskenazi B Bradman A Castorina R. Exposures of children to organophosphate pesticides and their potential adverse health effects. Environ Health Perspect (1999) 107:409–19. 10.1289/ehp.99107s3409 10346990 PMC1566222

[17] Jaeger JW Carlson IH Porter WP. Endocrine, immune, and behavioral effects of aldicarb (carbamate), atrazine (triazine) and nitrate (fertilizer) mixtures at groundwater concentrations. Toxicol Ind Health (1999) 15:133–51. 10.1177/074823379901500111 10188196

[18] García AM Pesticide exposure and women’s health. Am J Ind Med (2003) 44:584–94. 10.1002/ajim.10256 14635235

[19] Salameh P Waked M Baldi I Brochard P Saleh BA. Respiratory diseases and pesticide exposure: a case-control study in Lebanon. J Epidemiol Community Health (2006) 60:256–61. 10.1136/jech.2005.039677 16476757 PMC2465555

[20] Weisenburger DD. Human health effects of agrichemical use. Hum Pathol (1993) 24:571–6. 10.1016/0046-8177(93)90234-8 8505035

[21] Bradberry SM Proudfoot AT Vale JA. Glyphosate poisoning. Toxicol Rev (2004) 23:159–67. 10.2165/00139709-200423030-00003 15862083

[22] Bretveld RW Thomas CMG Scheepers PTJ Zielhuis GA Roeleveld N. Pesticide exposure: the hormonal function of the female reproductive system disrupted? Reprod Biol Endocrinol (2006) 4:30. 10.1186/1477-7827-4-30 16737536 PMC1524969

[23] Bassil KL Vakil C Sanborn M Cole DC Kaur JS Kerr KJ. Cancer health effects of pesticides. Systematic review. Can Fam Physician (2007) 53:1704–11. 17934034 PMC2231435

[24] Hoppin JA Umbach DM London SJ Henneberger PK Kullman GJ Alavanja MCR Pesticides and atopic and nonatopic asthma among farm women in the agricultural health study. Am J Respir Crit Care Med (2008) 177:11–8. 10.1164/rccm.200706-821OC 17932376 PMC2176117

[25] Bjørling-Poulsen M Andersen HR Grandjean P Potential developmental neurotoxicity of pesticides used in Europe. Environ Health (2008) 7:50 10.1186/1476-069X-7-50 18945337 PMC2577708

[26] Roeleveld N Bretveld R. The impact of pesticides on male fertility. Curr Opin Obstet Gynecol (2008) 20:229–33. 10.1097/GCO.0b013e3282fcc334 18460936

[27] Osman KA Pesticides and human health. In: Stoytcheva M, editor. Pesticides in the Modern World – Effects of Pesticides Exposure. InTech (2011). p. 206–30. Available from: http://www.intechopen.com/books/pesticides-in-the-modern-world-effects-of-pesticides-exposure/pesticides-and-human-health

[28] Mostafalou S Abdollahi M. Pesticides and human chronic diseases: evidences, mechanisms, and perspectives. Toxicol Appl Pharmacol (2013) 268:157–77. 10.1016/j.taap.2013.01.025 23402800

[29] Séralini GE Clair E Mesnage R Gress S Defarge N Malatesta M Republished study: long-term toxicity of a Roundup herbicide and a Roundup-tolerant genetically modified maize. Environ Sci Eur (2014) 26:14 10.1186/s12302-014-0014-5 PMC5044955 27752412

[30] Thakur DS Khot R Joshi PP Pandharipande M Nagpure K. Glyphosate poisoning with acute pulmonary edema. Toxicol Int (2014) 21:328–30. 10.4103/0971-6580.155389 25948977 PMC4413421

[31] Gunnell D Eddleston M Phillips MR Konradsen F. The global distribution of fatal pesticide self-poisoning: systematic review. BMC Public Health (2007) 7:357. 10.1186/1471-2458-7-357 18154668 PMC2262093

[32] McGill AE Robinson J Organochlorine insecticide residues in complete prepared meals: a 12-month survey in S.E. England. Food Cosmet Toxicol (1968) 6:45–57. 10.1016/0015-6264(68)90080-1 5761595

[33] Cabras P Angioni A. Pesticide residues in grapes, wine, and their processing products. J Agric Food Chem (2000) 48:967–73. 10.1021/jf990727a 10775335

[34] Zambonin CG Quinto M De Vietro N Palmisano F Solid-phase microextraction – gas chromatography mass spectrometry: a fast and simple screening method for the assessment of organophosphorus pesticides residues in wine and fruit juices. Food Chem (2004) 86:269–74. 10.1016/j.foodchem.2003.09.025

[35] Burnett M Welford R Case study: coca-cola and water in India: episode 2. Corp Soc Responsib Environ Mgmt (2007) 14:298–304. 10.1002/csr.97

[36] Lorenzin M. Pesticide residues in Italian ready-meals and dietary intake estimation. J Environ Sci Health B (2007) 42:823–33. 10.1080/03601230701555021 17763040

[37] Nag SK Raikwar MK Persistent organochlorine pesticides residues in animal feed. Environ Monit Assess (2011) 174:327–35. 10.1007/s10661-010-1460-1 20443138

[38] Witczak A Abdel-Gawad H. Assessment of health risk from organochlorine pesticides residues in high-fat spreadable foods produced in Poland. J Environ Sci Health B (2014) 49:917–28. 10.1080/03601234.2014.951574 25310807

[39] Chourasiya S Khillare PS Jyethi DS Health risk assessment of organochlorine pesticide exposure through dietary intake of vegetables grown in the periurban sites of Delhi, India. Environ Sci Pollut Res Int (2015) 22:5793–806. 10.1007/s11356-014-3791-x 25384696

[40] Reiler E Jørs E Bælum J Huici O Alvarez Caero MM Cedergreen N. The influence of tomato processing on residues of organochlorine and organophosphate insecticides and their associated dietary risk. Sci Total Environ (2015) 527–528:262–9. 10.1016/j.scitotenv.2015.04.081 25965039

[41] Nougadére A Sirot V Kadar A Fastier A Truchot E Vergnet C Total diet study on pesticide residues in France: levels in food as consumed and chronic dietary risk to consumers. Environ Int (2012) 45:135–50. 10.1016/j.envint.2012.02.001 22595191

[42] Blaznik U Yngve A Eržen I Hlastan Ribič C Consumption of fruits and vegetables and probabilistic assessment of the cumulative acute exposure to organophosphorus and carbamate pesticides of schoolchildren in Slovenia. Public Health Nutr (2015) 19(3):557–63. 10.1017/S1368980015001494 25990202 PMC10271107

[43] Kortenkamp A. Ten years of mixing cocktails: a review of combination effects of endocrine-disrupting chemicals. Environ Health Perspect (2007) 115:98–105. 10.1289/ehp.9357 18174957 PMC2174407

[44] Damgaard IN Skakkebaek NE Toppari J Virtanen HE Shen H Schramm KW Persistent pesticides in human breast milk and cryptorchidism. Environ Health Perspect (2006) 114:1133–8. 10.1289/ehp.8741 16835070 PMC1513324

[45] Buscail C Chevrier C Serrano T Pelé F Monfort C Cordier S Prenatal pesticide exposure and otitis media during early childhood in the PELAGIE mother-child cohort. Occup Environ Med (2015) 72(12):837–44. 10.1136/oemed-2015-103039 26347056

[46] Lu D Wang D Ni R Lin Y Feng C Xu Q Organochlorine pesticides and their metabolites in human breast milk from Shanghai, China. Environ Sci Pollut Res Int (2015) 22:9293–306. 10.1007/s11356-015-4072-z 25595932

[47] Baylis AD Why glyphosate is a global herbicide: strengths, weaknesses and prospects. Pest Manag Sci (2000) 56:299–308. 10.1002/(SICI)1526-4998(200004)56:4<299:AID-PS144>3.0.CO;2-K

[48] Turusov V Rakitsky V Tomatis L. Dichlorodiphenyltrichloroethane (DDT): ubiquity, persistence, and risks. Environ Health Perspect (2002) 110:125–8. 10.1289/ehp.02110125 11836138 PMC1240724

[49] Van den Berg H. Global status of DDT and its alternatives for use in vector control to prevent disease. Environ Health Perspect (2009) 117:1656–63. 10.1289/ehp.0900785 20049114 PMC2801202

[50] Rodríguez-Alcalá LM Sá C Pimentel LL Pestana D Teixeira D Faria A Endocrine disruptor DDE associated with a high-fat diet enhances the impairment of liver fatty acid composition in rats. J Agric Food Chem (2015) 63:9341–8. 10.1021/acs.jafc.5b03274 26449595

[51] Eskenazi B Marks AR Bradman A Fenster L Johnson C Barr DB In utero exposure to dichlorodiphenyltrichloroethane (DDT) and dichlorodiphenyldichloroethylene (DDE) and neurodevelopment among young Mexican American children. Pediatrics (2006) 118:233–41. 10.1542/peds.2005-3117 16818570

[52] Lemaire G Terouanne B Mauvais P Michel S Rahman R. Effect of organochlorine pesticides on human androgen receptor activation *in vitro*. Toxicol Appl Pharmacol (2004) 196:235–46. 10.1016/j.taap.2003.12.011 15081270

[53] Tiemann U. *In vivo* and *in vitro* effects of the organochlorine pesticides DDT, TCPM, methoxychlor, and lindane on the female reproductive tract of mammals: a review. Reprod Toxicol (2008) 25:316–26. 10.1016/j.reprotox.2008.03.002 18434086

[54] Karami-Mohajeri S Abdollahi M Toxic influence of organophosphate, carbamate, and organochlorine pesticides on cellular metabolism of lipids, proteins, and carbohydrates: a systematic review. Hum Exp Toxicol (2011) 30(9):1119–40. 10.1177/0960327110388959 21071550

[55] Freire C Koifman RJ Koifman S. Hematological and hepatic alterations in brazilian population heavily exposed to organochlorine pesticides. J Toxicol Environ Health A (2015) 78:534–48. 10.1080/15287394.2014.999396 25849770

[56] Calle EE Frumkin H Henley SJ Savitz DA Thun MJ. Organochlorines and breast cancer risk. CA Cancer J Clin (2002) 52:301–9. 10.3322/canjclin.52.5.301 12363327

[57] Robinson T Ali U Mahmood A Chaudhry MJ Li J Zhang G Concentrations and patterns of organochlorines (OCs) in various fish species from the Indus River, Pakistan: a human health risk assessment. Sci Total Environ (2015) 541:1232–42. 10.1016/j.scitotenv.2015.10.002 26476063

[58] Jaga K Dharmani C. Sources of exposure to and public health implications of organophosphate pesticides. Rev Panam Salud Publica (2003) 14:171–85. 10.1590/S1020-49892003000800004 14653904

[59] McKinlay R Plant JA Bell JNB Voulvoulis N. Endocrine disrupting pesticides: implications for risk assessment. Environ Int (2008) 34:168–83. 10.1016/j.envint.2007.07.013 17881056

[60] Gasnier C Dumont C Benachour N Clair E Chagnon MC Séralini GE. Glyphosate-based herbicides are toxic and endocrine disruptors in human cell lines. Toxicology (2009) 262:184–91. 10.1016/j.tox.2009.06.006 19539684

[61] Li D Huang Q Lu M Zhang L Yang Z Zong M The organophosphate insecticide chlorpyrifos confers its genotoxic effects by inducing DNA damage and cell apoptosis. Chemosphere (2015) 135:387–93. 10.1016/j.chemosphere.2015.05.024 26002045

[62] Hung DZ Yang HJ Li YF Lin CL Chang SY Sung FV The long-term effects of organophosphates poisoning as a risk factor of CVDs: a nationwide population-based cohort study. PLoS One (2015) 10:e0137632. 10.1371/journal.pone.0137632 26339906 PMC4560399

[63] Jamal F Haque QS Singh S Rastogi S. The influence of organophosphate and carbamate on sperm chromatin and reproductive hormones among pesticide sprayers. Toxicol Ind Health (2015):1–10. 10.1177/0748233714568175 25647813

[64] Rosenstock L Keifer M Daniell WE McConnell R Claypoole K Chronic central nervous system effects of acute organophosphate pesticide intoxication. Lancet (1991) 338:223–7. 10.1016/0140-6736(91)90356-T 1676786

[65] Wesseling C Keifer M Ahlbom A McConnell R Moon J-D Rosenstock L Long-term neurobehavioral effects of mild poisonings with organophosphate and n-methyl carbamate pesticides among banana workers. Int J Occup Environ Health (2002) 8:27–34. 10.1179/oeh.2002.8.1.27 11843437

[66] Eskenazi B Harley K Bradman A Fenster L Wolff M Engel S In utero pesticide exposure and neurodevelopment in three NIEHS/environmental agency children’s center birth cohorts. Epidemiology (2006) 17:S103 10.1097/00001648-200611001-00249

[67] Lin JN Lin CL Lin MC Lai CH Lin HH Yang CH Increased risk of dementia in patients with acute organophosphate and carbamate poisioning: a nationwide population-based cohort study. Medicine (Baltimore) (2015) 94:e1187 10.1097/MD.0000000000001187 26200627 PMC4603014

[68] Waddell BL Zahm SH Baris D Weisenburger DD Holmes F Burmeister LF Agricultural use of organophosphate pesticides and the risk of non-Hodgkin’s lymphoma among male farmers (United States). Cancer Causes Control (2001) 12:509–17. 10.1023/A:1011293208949 11519759

[69] Eskenazi B Harley K Bradman A Weltzien E Jewel NP Barr DB Association of *in Utero* organophosphate pesticide exposure and fetal growth and length of gestation in an agricultural population. Environ Health Perspect (2004) 112:1116–24. 10.1289/ehp.6789 15238287 PMC1247387

[70] Rauh VA Garcia WE Whyatt RM Horton MK Barr DB Louis ED. Prenatal exposure to the organophosphate pesticide chlorpyrifos and childhood tremor. Neurotoxicology (2015) 51:80–6. 10.1016/j.neuro.2015.09.004 26385760 PMC4809635

[71] Williams GM Kroes R Munro IC. Safety evaluation and risk assessment of the herbicide Roundup and its active ingredient, glyphosate, for humans. Regul Toxicol Pharmacol (2000) 31:117–65. 10.1006/rtph.1999.1371 10854122

[72] Mink PJ Mandel JS Lundin JI Sceurman BK Epidemiologic studies of glyphosate and non-cancer health outcomes. Regul Toxicol Pharmacol (2011) 61:172–84. 10.1016/j.yrtph.2011.07.006 21798302

[73] Mink PJ Mandel JS Sceurman BK Lundin JI. Epidemiologic studies of glyphosate and cancer: a review. Regul Toxicol Pharmacol (2012) 63:440–52. 10.1016/j.yrtph.2012.05.012 22683395

[74] Cuhra M Traavik T Bøhn T. Clone- and age-dependent toxicity of a glyphosate commercial formulation and its active ingredient in Daphnia magna. Ecotoxicology (2013) 22:251–62. 10.1007/s10646-012-1021-1 23224423 PMC3572389

[75] Campbell AW Glyphosate: its effects on humans. Altern Ther Health Med (2014) 20:9–11. 24755565

[76] Samsel A Seneff S Glyphosate’s suppression of cytochrome P450 enzymes and amino acid biosynthesis by the gut microbiome: pathways to modern diseases. Entropy (2013) 15:1416–63. 10.3390/e15041416

[77] Woodburn AT Glyphosate: production, pricing and use worldwide. Pest Manag Sci (2000) 56:309–12. 10.1002/(SICI)1526-4998(200004)

[78] Bonny S Genetically modified glyphosate-tolerant soybean in the USA: adoption factors, impacts and prospects. A review. Agron Sustain Dev (2008) 28:21–32. 10.1051/agro:2007044

[79] Duke SO Powles SB Glyphosate-resistant crops and weeds: now and in the future. AgBioForum (2009) 12:346–57.

[80] Thongprakaisang S Thiantanawat A Rangkadilok N Suriyo T Satayavivad J. Glyphosate induces human breast cancer cells growth via estrogen receptors. Food Chem Toxicol (2013) 59:129–36. 10.1016/j.fct.2013.05.057 23756170

[81] Fukutake M Takahashi M Ishida K Kawamura H Sugimura T Wakabayashi K. Quantification of genistein and genistin in soybeans and soybean products. Food Chem Toxicol (1996) 34:457–61. 10.1016/0278-6915(96)87355-8 8655094

[82] Swanson NL Leu A Abrahamson J Wallet B Genetically engineered crops, glyphosate and the deterioration of health in the United States of America. J Org Syst (2014) 9:6–37.

[83] Kwiatkowska M Huras B Bukowska B. The effect of metabolites and impurities of glyphosate on human erythrocytes (*in vitro*). Pestic Biochem Physiol (2014) 109:34–43. 10.1016/j.pestbp.2014.01.003 24581382

[84] George J Prasad S Mahmood Z Shukla Y. Studies on glyphosate-induced carcinogenicity in mouse skin: a proteomic approach. J Proteomics (2010) 73:951–64. 10.1016/j.jprot.2009.12.008 20045496

[85] Samsel A Seneff S. Glyphosate, pathways to modern diseases III: manganese, neurological diseases, and associated pathologies. Surg Neurol Int (2015) 6:45. 10.4103/2152-7806.153876 25883837 PMC4392553

[86] Pieniażek D Bukowska B Duda W Comparison of the effect of Roundup Ultra 360 SL pesticide and its active compound glyphosate on human erythrocytes. Pestic Biochem Physiol (2004) 79:58–63. 10.1016/j.pestbp.2004.03.003

[87] Kwiatkowska M Pawel J Bukowska B Glyphosate and its formulations – toxicity, occupational and environmental exposure (Article in Polish). Med Pr (2013) 64:717–29. 10.13075/mp.5893.2013.0059 24502134

[88] Clair É Mesnage R Travert C Séralini G-É. A glyphosate-based herbicide induces necrosis and apoptosis in mature rat testicular cells *in vitro*, and testosterone decrease at lower levels. Toxicol In Vitro (2012) 26:269–79. 10.1016/j.tiv.2011.12.009 22200534

[89] Gehin A Guyon C Nicod L. Glyphosate-induced antioxidant imbalance in HaCaT: the protective effect of Vitamins C and E. Environ Toxicol Pharmacol (2006) 22:27–34. 10.1016/j.etap.2005.11.003 21783682

[90] de Liz Oliveira Cavalli VL Cattani D Heinz Rieg CE Pierozan P Zanatta L Benedetti Parisotto E Roundup disrupts male reproductive functions by triggering calcium-mediated cell death in rat testis and Sertoli cells. Free Radic Biol Med (2013) 65:335–46. 10.1016/j.freeradbiomed.2013.06.043 23820267

[91] Hedberg D Wallin M. Effects of Roundup and glyphosate formulations on intracellular transport, microtubules and actin filaments in *Xenopus laevis* melanophores. Toxicol In Vitro (2010) 24:795–802. 10.1016/j.tiv.2009.12.020 20036731

[92] Samsel A Seneff S. Glyphosate, pathways to modern diseases II: celiac sprue and gluten intolerance. Interdiscip Toxicol (2013) 6:159–84. 10.2478/intox-2013-0026 24678255 PMC3945755

[93] Goad ER Goad JT Atieh BH Gupta RC. Carbofuran-induced endocrine disruption in adult male rats. Toxicol Mech Methods (2004) 14:233–9. 10.1080/15376520490434476 20021136

[94] Soloneski S Kujawski M Scuto A Larramendy ML. Carbamates: a study on genotoxic, cytotoxic, and apoptotic effects induced in Chinese hamster ovary (CHO-K1) cells. Toxicol In Vitro (2015) 29:834–44. 10.1016/j.tiv.2015.03.011 25820133

[95] Li Q Kobayashi M Kawada T. Ziram induces apoptosis and necrosis in human immune cells. Arch Toxicol (2011) 85:355–61. 10.1007/s00204-010-0586-9 20842346

[96] Li Q Kobayashi M Kawada T Mechanism of ziram-induced apoptosis in human natural killer cells. Int J Immunopathol Pharmacol (2012) 25:883–91. 10.1177/039463201202500406 23298479

[97] Li Q Kobayashi M Kawada T. Carbamate pesticide-induced apoptosis and necrosis in human natural killer cells. J Biol Regul Homeost Agents (2014) 28:23–32. 24750788

[98] Li Q Kobayashi M Kawada T. Carbamate pesticide-induced apoptosis in human T lymphocytes. Int J Environ Res Public Health (2015) 12:3633–45. 10.3390/ijerph120403633 25837344 PMC4410207

[99] Denison MS Phelan D Winter GM Ziccardi MH. Carbaryl, a carbamate insecticide, is a ligand for the hepatic ah (dioxin) receptor. Toxicol Appl Pharmacol (1998) 152:406–14. 10.1006/taap.1998.9999 9853009

[100] Lifshitz M Shahak E Bolotin A Sofer S. Carbamate poisoning in early childhood and in adults. J Toxicol Clin Toxicol (1997) 35:25–7. 10.3109/15563659709001161 9022648

[101] Zheng T Zahm SH Cantor KP Weisenburger DD Zhang Y Blair A. Agricultural exposure to carbamate pesticides and risk of non-hodgkin lymphoma. J Occup Environ Med (2001) 43:641–9. 10.1097/00043764-200107000-00012 11464396

[102] Kniewald J Jakominić M Tomljenović A Simić B Romać P Vranesić D Disorders of male rat reproductive tract under the influence of atrazine. J Appl Toxicol (2000) 20:61–8. 10.1002/(SICI)1099-1263(200001/02)20:1<61:AID-JAT628>3.0.CO;2-3 10641017

[103] Jin Y Wang L Chen G Lin X Miao W Fu Z. Exposure of mice to atrazine and its metabolite diaminochlorotriazine elicits oxidative stress and endocrine disruption. Environ Toxicol Pharmacol (2014) 37:782–90. 10.1016/j.etap.2014.02.014 24632104

[104] Kettles MK Browning SR Prince TS Horstman SW. Triazine herbicide exposure and breast cancer incidence: an ecologic study of Kentucky counties. Environ Health Perspect (1997) 105:1222–7. 10.1289/ehp.971051222 9370519 PMC1470339

[105] Liu XM Shao JZ Xiang LX Chen XY. Cytotoxic effects and apoptosis induction of atrazine in a grass carp (*Ctenopharyngodon idellus*) cell line. Environ Toxicol (2006) 21:80–9. 10.1002/tox.20159 16463256

[106] Huang P Yang J Song Q. Atrazine affects phosphoprotein and protein expression in MCF-10A human breast epithelial cells. Int J Mol Sci (2014) 15:17806–26. 10.3390/ijms151017806 25275270 PMC4227191

[107] Li YS He X Ma K Wu YP Li BX. The effect of exposure to atrazine on dopaminergic development in pubertal male SD rats. Birth Defects Res B Dev Reprod Toxicol (2015) 104:184–9. 10.1002/bdrb.21151 26331294

[108] Ma K Wu HY Zhang B He X Li BX. Neurotoxicity effects of atrazine-induced SH-SY5Y human dopaminergic neuroblastoma cells via microglial activation. Mol Biosyst (2015) 11:2915–24. 10.1039/c5mb00432b 26256823

[109] Song Y Jia ZC Chen JY Hu JX Zhang LS. Toxic effects of atrazine on reproductive system of male rats. Biomed Environ Sci (2014) 27:281–8. 10.3967/bes2014.050 24758756

[110] Breckenridge CB Sawhney Coder P Tisdel MO Simpkins JW Yi KD Foradori CD Effect of age, duration of exposure, and dose of atrazine on sexual maturation and the luteinizing hormone surge in the female Sprague-Dawley rat. Birth Defects Res B Dev Reprod Toxicol (2015) 104:204–17. 10.1002/bdrb.21154 26439775 PMC4992940

[111] Kolaczinski JH Curtis CF. Chronic illness as a result of low-level exposure to synthetic pyrethroid insecticides: a review of the debate. Food Chem Toxicol (2004) 42:697–706. 10.1016/j.fct.2003.12.008 15046814

[112] Ray DE Fry JR A reassessment of the neurotoxicity of pyrethroid insecticides. Pharmacol Ther (2006) 111:174–93. 10.1016/j.pharmthera.2005.10.003 16324748

[113] Garey J Wolff MS. Estrogenic and antiprogestagenic activities of pyrethroid insecticides. Biochem Biophys Res Commun (1998) 251:855–9. 10.1006/bbrc.1998.9569 9790999

[114] Jaensson A Scott AP Moore A Kylin H Håkan Olsén K. Effects of a pyrethroid pesticide on endocrine responses to female odours and reproductive behaviour in male parr of brown trout (*Salmo trutta* L.). Aquat Toxicol (2007) 81:1–9. 10.1016/j.aquatox.2006.10.011 17174415

[115] Pandey SP Mohanty B The neonicotinoid pesticide imidacloprid and the dithiocarbamate fungicide mancozeb disrupt the pituitary–thyroid axis of a wildlife bird. Chemosphere (2014) 122:227–34. 10.1016/j.chemosphere.2014.11.061 25496744

[116] Moore A Waring CP. The effects of a synthetic pyrethroid pesticide on some aspects of reproduction in Atlantic salmon (*Salmo salar* L.). Aquat Toxicol (2001) 52:1–12. 10.1016/S0166-445X(00)00133-8 11163426

[117] Jurewicz J Radwan M Wielgomas B Sobala W Piskunowicz M Radwan P The effect of environmental exposure to pyrethroids and DNA damage in human sperm. Syst Biol Reprod Med (2015) 61:37–43. 10.3109/19396368.2014.981886 25376306

[118] Shafer TJ Meyer DA Crofton KM. Developmental neurotoxicity of pyrethroid insecticides: critical review and future research needs. Environ Health Perspect (2005) 113:123–36. 10.1289/ehp.7254 15687048 PMC1277854

[119] Syed F John PJ Soni I. Neurodevelopmental consequences of gestational and lactational exposure to pyrethroids in rats. Environ Toxicol (2015). 10.1002/tox.22178 26460727

[120] Goulson D An overview of the environmental risks posed by neonicotinoid insecticides. J Appl Ecol (2013) 50:977–87. 10.1111/1365-2664.12111

[121] Jeschke P Nauen R Neonicotinoids – from zero to hero in insecticide chemistry. Pest Manag Sci (2008) 64:1084–98. 10.1002/ps.1631 18712805

[122] Qi S Wang C Chen X Qin Z Li X Wang C. Toxicity assessments with Daphnia magna of Guadipyr, a new neonicotinoid insecticide and studies of its effect on acetylcholinesterase (AChE), glutathione S-transferase (GST), catalase (CAT) and chitobiase activities. Ecotoxicol Environ Saf (2013) 98:339–44. 10.1016/j.ecoenv.2013.09.013 24075643

[123] Put K Bollens T Wäckers F Pekas A Non-target effects of commonly used plant protection products in roses on the predatory mite *Euseius gallicus* Kreiter & Tixier (Acari: Phytoseidae). Pest Manag Sci (2016) 72(7):1373–80. 10.1002/ps.4162 26434923

[124] Williams GR Troxler A Retschnig G Roth K Yañez O Shutler D Neonicotinoid pesticides severely affect honey bee queens. Sci Rep (2015) 5:14621. 10.1038/srep14621 26459072 PMC4602226

[125] Wright GA Softley S Earnshaw H. Low doses of neonicotinoid pesticides in food rewards impair short-term olfactory memory in foraging-age honeybees. Sci Rep (2015) 5:15322. 10.1038/srep15322 26477973 PMC4609922

[126] Bal R Naziroğlu M Türk G Yilmaz Ö Kuloğlu T Etem E Insecticide imidacloprid induces morphological and DNA damage through oxidative toxicity on the reproductive organs of developing male rats. Cell Biochem Funct (2012) 30:492–9. 10.1002/cbf.2826 22522919

[127] Hoshi N Hirano T Omotehara T Tokumoto J Umemura Y Mantani Y. Insight into the mechanism of reproductive dysfunction caused by neonicotinoid pesticides. Biol Pharm Bull (2014) 37:1439–43. 10.1248/bpb.b14-00359 25177026

[128] Caron-Beaudoin É Denison MS Sanderson JT Effects of neonicotinoids on promoter-specific expression and activity of aromatase (CYP19) in human adrenocortical carcinoma (H295R) and primary umbilical vein endothelial (HUVEC) cells. Toxicol Sci (2016) 149(1):134–44. 10.1093/toxsci/kfv220 26464060

[129] Pierce FJ Nowak P Aspects of precision agriculture. Adv Agron (1999) 67:1–85. 10.1016/S0065-2113(08)60513-1

[130] Robert PC Precision agriculture: a challenge for crop nutrition management. Plant Soil (2002) 247:143–9. 10.1023/A:1021171514148

[131] Zhang N Wang M Wang N Precision agriculture – a worldwide overview. Compu Electron Agr (2002) 36:113–32. 10.1016/S0168-1699(02)00096-0

[132] Bongiovanni R Lowenberg-DeBoer J Precision agriculture and sustainability. Precision Agric (2004) 5:359–87. 10.1023/B:PRAG.0000040806.39604.aa

[133] McBratney A Whelan B Ancev T Bouma J Future direction of precision agriculture. Precision Agric (2005) 6:7–23. 10.1007/s11119-005-0681-8

[134] Watcharaanantapong P Roberts RK Lambert DM Larson JA Velandia M English BC Timing of precision agriculture technology adoption in US cotton production. Precision Agric (2014) 15:427–46. 10.1007/s11119-013-9338-1

[135] Zhang C Kovacs JM The application of small unmanned aerial systems for precision agriculture: a review. Precision Agric (2012) 13:693–712. 10.1007/s11119-012-9274-5

[136] Hertoge K Mals/Malles Venosta Referendum. (2014). Available from: http://www.marcozullo.it/wp-content/uploads/Malles-Venosta-Referendum.pdf

[137] Menezes F Food sovereignty: a vital requirement for food security in the context of globalization. Development (2001) 44:29–33. 10.1057/palgrave.development.1110288

[138] Windfuhr M Jonsén J Food Sovereignty. Towards Democracy in Localized Food Systems. Chippenham, GB: ITDG Publishing (2005).

[139] Declaration of Nyéléni. (2007). Available from: http://nyeleni.org/spip.php?article290

[140] Patel R Food sovereignty. J Peasant Stud (2009) 36:663–706. 10.1080/03066150903143079

[141] Wittman H Food sovereignty: a new rights framework for food and nature? Environ Soc Adv Res (2011) 2:87–105. 10.3167/ares.2011.020106

[142] Pimbert M Towards Food Sovereignty: Reclaiming Autonomous Food Systems. London, Munich: CAFS, IIED, RCC (2009). Available from: http://pubs.iied.org/pdfs/G02268.pdf

[143] Altieri MA Agroecology, small farms, and food sovereignty. Mon Rev (2009) 61:102–13. 10.14452/MR-061-03-2009-07_8

[144] Holt-Giménez E From food crisis to food sovereignty. The challenge of social movements. Mon Rev (2009) 61:142–56. 10.14452/MR-061-03-2009-07_11

[145] Rosset P Food sovereignty and the contemporary food crisis. Development (2008) 51:460–3. 10.1057/dev.2008.48

[146] Panagiotakopulu E Buckland PC Day PM Sarpaki AA Doumas C Natural insecticides and insect repellents in antiquity: a review of the evidence. J Archaeol Sci (1995) 22:705–10. 10.1016/S0305-4403(95)80156-1

[147] Fry DM. Reproductive effects in birds exposed to pesticides and industrial chemicals. Environ Health Perspect (1995) 103:165–71. 10.2307/3432528 8593865 PMC1518881

[148] Shukla G Kumar A Bhanti M Joseph PE Taneja A. Organochlorine pesticide contamination of ground water in the city of Hyderabad. Environ Int (2006) 32:244–7. 10.1016/j.envint.2005.08.027 16183122

[149] Hemingway J Ranson H. Insecticide resistance in insect vectors of human disease. Annu Rev Entomol (2000) 45:371–91. 10.1146/annurev.ento.45.1.371 10761582

[150] Olesen JE Bindi M Consequences of climate change for European agricultural productivity, land use and policy. Eur J Agron (2002) 16:239–62. 10.1016/S1161-0301(02)00004-7

[151] Taiz L Agriculture, plant physiology, and human population growth: past, present, and future. Theor Exp Plant Physiol (2013) 25:167–81. 10.1590/S2197-00252013000300001

[152] Steenwerth KL Hodson AK Bloom AJ Carter MR Cattaneo A Chartres CJ Climate-smart agriculture global research agenda: scientific basis for action. Agr Food Secur (2014) 3:11 10.1186/2048-7010-3-11

[153] Tissier J Grosclaude JY What about climate-smart agriculture? In: Torquebiau E, editor. Climate Change and Agriculture Worldwide. Dordrecht, Heidelberg, New York, London: Springer Science + Business Media Dordrecht (2016). p. 313–24.

[154] McIntyre BD Herren HR Wakhungu J Watson RT, editors. Agriculture at a Crossroads. IAASTD Global Report. Washington, Covelo, London: Island Press, IAASTD (2009). Available from: http://www.unep.org/dewa/agassessment/reports/IAASTD/EN/Agriculture%20at%20a%20Crossroads_Global%20Report%20(English).pdf

[155] UNCTAD, UNEP. Organic Agriculture and Food Security in Africa. New York, Geneva: United Nations (2008). Available from: http://unctad.org/en/docs/ditcted200715_en.pdf

[156] Dona A Arvanitoyannis IS. Health risks of genetically modified foods. Crit Rev Food Sci Nutr (2009) 49:164–75. 10.1080/10408390701855993 18989835

[157] McHughen A. GM crops and foods. What do consumers want to know? GM Crops Food (2013) 4:172–82. 10.4161/gmcr.26532 24051491

[158] Benbrook C Do GM crops mean less pesticide use? Pestic Outlook (2001) 12:204–7. 10.1039/b108609j

[159] Huang G Hu R Pray C Qiao F Rozelle S Biotechnology as an alternative to chemical pesticides: a case study of Bt cotton in China. Agr Econ (2003) 29:55–67. 10.1016/S0169-5150(03)00044-6

[160] Benbrook CM Impacts of genetically engineered crops on pesticide use in the U.S. – the first sixteen years. Environ Sci Eur (2012) 24:24 10.1186/2190-4715-24-24

[161] Sexton K. Cumulative risk assessment: an overview of methodological approaches for evaluating combined health effects from exposure to multiple environmental stressors. Int J Environ Res Public Health (2012) 9:370–90. 10.3390/ijerph9020370 22470298 PMC3315252

[162] Colosio C Alegakis AK Tsatsakis AM Emerging health issues from chronic pesticide exposure: innovative methodologies and effects on molecular cell and tissue level. Toxicology (2013) 307:1–2. 10.1016/j.tox.2013.04.006 23664459

[163] Hernández AF Parrón T Tsatsakis AM Requena M Alarcón R López-Guarnido O. Toxic effects of pesticide mixtures at a molecular level: their relevance to human health. Toxicology (2013) 307:136–45. 10.1016/j.tox.2012.06.009 22728724

[164] Pimentel D Burgess M Environmental and economic benefits of reducing pesticide use. In: Pimentel D Peshin R, editors. Integrated Pest Management. New York, Heidelberg, Dordrecht, London: Springer Science + Business Media Dordrecht (2014). p. 127–39.

[165] European Commission. The Common Agricultural Policy: A partnership between Europe and Farmers. Luxembourg: Publications Office of the European Union (2012). Available from: http://ec.europa.eu/agriculture/cap-overview/2012_en.pdf

